# Endometrial Atypical Hyperplasia and Risk of Endometrial Cancer

**DOI:** 10.3390/diagnostics14222471

**Published:** 2024-11-05

**Authors:** An-Ju Chou, Ruo-Shi Bing, Dah-Ching Ding

**Affiliations:** 1 Department of Medicine, Taipei Tzu Chi Hospital, Buddhist Tzu Chi Medical Foundation, Tzu Chi University, New Taipei City 231, Taiwan; whoisright666@gmail.com (A.-J.C.); rosebing94@gmail.com (R.-S.B.); 2 Department of Obstetrics and Gynecology, Hualien Tzu Chi Hospital, Buddhist Tzu Chi Medical Foundation, Tzu Chi University, Hualien 970, Taiwan; 3 Institute of Medical Sciences, Tzu Chi University, Hualien 970, Taiwan

**Keywords:** endometrial cancer, endometrial atypical hyperplasia, surgery, differential diagnosis, fertility sparing

## Abstract

Endometrial atypical hyperplasia (EAH) is a premalignant condition with a substantial risk of progression to endometrial cancer (EC), with the endometrioid subtype being the most common. EAH is characterized by abnormal endometrial gland proliferation and cellular atypia, often resulting from prolonged unopposed estrogen exposure. This review aims to explore the clinical significance of EAH, its risk of progression to EC, and the current approaches to management. The risk of EAH progressing to EC ranges from 20 to 50%, influenced by factors such as histopathology and genetic mutations including *PTEN* and *KRAS*. Key risk factors include obesity, polycystic ovary syndrome, and postmenopausal status. Abnormal uterine bleeding is a hallmark symptom of EAH and early-stage EC, necessitating diagnostic evaluation through endometrial biopsy and transvaginal ultrasonography. Therapeutic management strategies depend on patient risk and fertility considerations. Hormonal therapy, particularly progestins, is the mainstay for fertility preservation, while hysterectomy is preferred for higher-risk patients. Regular monitoring with biopsies is essential for those undergoing conservative treatment. Recent advancements in the management of EAH and EC have shifted towards incorporation of molecular diagnostics and targeted therapies, enabling better risk stratification and individualized care. Biomarkers and minimally invasive surgical techniques are emerging as promising approaches in improving outcomes for women with EAH. This review underscores the importance of early diagnosis and personalized management in preventing the progression of EAH to EC, highlighting current clinical practices and potential future developments in this field.

## 1. Introduction

Endometrial atypical hyperplasia (EAH) is a precancerous condition of the endometrium, the inner lining of the uterus [1]. It is characterized by abnormal proliferation of endometrial glandular cells with atypical features, such as enlarged nuclei, irregular cell shapes, and loss of normal glandular architecture [2]. These cellular changes differentiate EAH from benign endometrial hyperplasia, where the glandular cells are proliferative but lack atypical characteristics [3].

EAH is more frequently observed in postmenopausal women and/or premenopausal women with risk factors such as obesity, unopposed estrogen exposure, polycystic ovary syndrome (PCOS), or a history of infertility [4]. Clinically, it presents with abnormal uterine bleeding, but in some cases, it may be asymptomatic and detected only through endometrial biopsy [5].

Within the spectrum of endometrial diseases, EAH stands out as a key precursor to more severe pathologies [6]. On one end of the spectrum, benign endometrial hyperplasia involves an overgrowth of normal endometrial tissue, while on the other, EC represents a full progression to malignancy. EAH occupies an intermediate stage, where the cellular changes carry a significant risk for malignant transformation, especially in the presence of persistent hormonal imbalances [2].

The importance of studying EAH lies in its strong association with endometrial cancer (EC), particularly type I endometrioid adenocarcinoma [7,8]. Type I endometrial cancer, which accounts for the majority of ECs, has a significant overlap of risk factors with EAH, including obesity, chronic estrogen stimulation, and metabolic syndromes [9].

EAH has been identified as a direct precursor to type I endometrioid adenocarcinoma, with studies revealing that up to 40–50% of women diagnosed with EAH who do not receive effective therapy may develop EC [10]. This progression makes EAH a crucial condition for early diagnosis and intervention [11]. The potential to halt or reverse the progression from EAH to malignancy with timely chemotherapeutic or surgical intervention highlights the clinical significance of EAH in reducing EC incidence [11].

By understanding the pathology and risk factors associated with EAH, clinicians and researchers may more effectively identify at-risk patients, develop preventive strategies, and improve patient outcomes.

### Study Research Strategy

The study involved systematically searching with the keywords “endometrial atypical hyperplasia, endometrial cancer” from their respective inception to 31 August 2024. Synonyms and derivatives of keywords were also used. The bibliographies of relevant reviews and included studies were also scrutinized. Table 1 describes the search strategy used for the PubMed database.

## 2. Pathophysiology of Endometrial Atypical Hyperplasia

### 2.1. Histological Features

EAH is distinguished by specific histological features that reflect the abnormal growth and atypical nature of the endometrial cells [12]. Key histological features include the following points.

- Glandular Crowding: In EAH, there is a marked increase in the number and density of endometrial glands, which also assume irregular shapes. This glandular crowding is accompanied by a loss of the normal gland-to-stroma ratio, as there is an overgrowth of glandular elements relative to the supportive stromal tissue [13].
- Nuclear Atypia: A hallmark of atypical hyperplasia is the presence of nuclear atypia, where the glandular cells exhibit nuclear enlargement, irregular nuclear contours, increased nuclear-to-cytoplasmic ratios, and chromatin clumping. This shift is a critical indicator of disease progression, as it reflects uncontrolled glandular growth suggesting an increased potential for malignancy [13].
- Cellular Architecture: The cellular arrangement is often irregular, with complex, maze-like configurations. There is a disruption in the usual orderly arrangement of cells, with back-to-back glands forming a complex pattern. Loss of cellular polarity is also seen, where the orientation of cells becomes disorganized [13,14].

### 2.2. Molecular Changes and Genetic Pathways

The progression of EAH to EC is driven by a series of molecular and genetic alterations [15]. Some of the key molecular changes implicated in this transformation include the following points.

- *PTEN* mutations: One of the most common genetic abnormalities associated with both EAH and type I endometrial carcinoma is the loss of function of the tumor suppressor gene phosphatase and tensin homolog (*PTEN*) [15]. *PTEN* mutations lead to hyperactivation of the phosphatidylinositol 3-kinase/protein kinase B (PI3K/AKT) pathway, which promotes cell proliferation and survival [15]. Loss of PTEN function is seen in approximately 55–80% of EAH cases and is considered an early event in endometrial carcinogenesis [16,17].
- *KRAS* Mutations: Mutations in the Kirsten rat sarcoma viral oncogene homolog (*KRAS*) gene, which encodes a protein involved in regulating cell growth and division, are also commonly found in EAH [18,19,20]. These mutations activate signaling pathways that promote cellular proliferation, contributing to the progression from hyperplasia to carcinoma. *KRAS* mutations are detected in about 10–30% of endometrial hyperplasia cases.
- Microsatellite Instability (MSI): MSI is a form of genetic hypermutability resulting from impaired DNA mismatch repair [21]. This abnormality is observed in a subset of EAH cases and is associated with an increased risk of progression to EC. MSI is also frequently observed in Lynch syndrome-associated ECs [22,23].
- Hormonal Imbalances: Unopposed estrogen exposure is a key factor in the development of EAH and its progression to cancer [24,25]. Estrogen stimulates the proliferation of endometrial cells, while progesterone acts by counterbalancing through promotion of cellular differentiation and apoptosis [26]. In cases of prolonged estrogen exposure without sufficient progesterone such as in obesity, PCOS, or anovulatory cycles, the endometrium undergoes unchecked proliferation, increasing the risk of hyperplasia and subsequent neoplastic transformation [27,28]. This hormonal imbalance is a common feature in both EAH and type I endometrioid carcinoma.

### 2.3. Progression to Endometrial Cancer

A substantial risk exists for progression from EAH to EC, making early detection and treatment crucial. Studies suggest that approximately 20–50% untreated cases of EAH progress to EC, specifically type I endometrioid adenocarcinoma [29,30]. The wide range in risk estimates is likely due to differences in patient populations, risk factors, and study methodologies.

- 20–25% Progression Risk: Lower estimates of progression risk are seen in some studies, particularly among patients with a short duration of untreated hyperplasia or those undergoing close surveillance and management [31]. Women with atypical endometrial hyperplasia have a 32.6% (95% CI: 24.1%, 42.4%) prevalence of concurrent endometrial cancer and an 8.2% (95% CI 3.9%, 17.3%) annual incidence rate of progression to cancer [32]. For EAH, the cumulative risk of progression rose from 8.2% (95% CI, 1.3–14.6%) at 4 years, to 12.4% (95% CI, 3.0–20.8%) at 9 years, and reached 27.5% (95% CI, 8.6–42.5%) at 19 years following diagnosis [29]. The cumulative 20-year progression risk from EAH to carcinoma is 28% [29]. EAH has a 23% risk of progression to carcinoma, compared to 1.6% for hyperplasia without atypia [31]. The study found a 45.9% rate of endometrial carcinoma in women with a biopsy diagnosis of atypical endometrial hyperplasia [33]. The cumulative 20-year progression risk for EAH is estimated at 27.5%, compared to less than 5% for simple EH [34]. For atypical endometrial polyps, the pooled risk estimate of concurrent EC is 5.6%, lower than the 42% risk associated with non-polypoid atypical EH [35].

The World Health Organization (WHO) system, which considers cytologic atypia, shows a relative risk (RR) of 8.74 for cancer progression, while the EAH with D-score demonstrates a significantly higher RR of 29.22 [36]. Complex hyperplasia without atypia increases cancer risk by 4.90 times compared to simple EH [37]. Women aged ≤39 and ≥50 years with EH have a higher risk of EC progression, but multiple follow-up biopsies increase EC detection [38].

- Progression Risk of 40–50%: Higher estimates are observed in patients with significant risk factors, such as obesity, prolonged unopposed estrogen exposure, and untreated chronic hyperplasia [39]. In such cases, the odds of malignant transformation are substantial. Exogenous estrogen therapy is associated with a 2.5-fold increased risk of endometrial cancer [40]. Nine out of twelve studies demonstrated a significantly increased risk of EC among users of estrogen-only menopausal hormone therapy, with odds ratios/hazard ratios ranging from 1.45 to 4.46 [41].

## 3. Risk Factors for EAH and Cancer Progression

### 3.1. Hormonal Imbalance

One of the most critical drivers of EAH and its progression to EC is unopposed estrogen exposure, where the endometrium is subjected to prolonged estrogenic stimulation without the compensating effects of progesterone [2]. This hormonal imbalance leads to disproportionate proliferation of endometrial cells, increasing the likelihood of atypical hyperplasia and subsequent malignancy.

Key factors contributing to hormonal imbalances include the following points.

- Obesity: Adipose tissue is a significant source of estrogen production in postmenopausal women [42]. Obesity leads to increased peripheral conversion of androgens to estrogens in adipose tissue, mediated by the enzyme aromatase [43]. This excessive estrogen is not balanced by progesterone, especially in postmenopausal women or those with anovulatory cycles, promoting endometrial proliferation [44]. Obese women have a 2–4-fold higher risk of developing endometrial hyperplasia and cancer compared to women with normal weight [45].
- PCOS: PCOS is characterized by chronic anovulation, where women often experience irregular menstrual cycles or may not ovulate at all, resulting in prolonged exposure to estrogen without the balancing effect of progesterone [46]. This hormonal environment increases the risk of endometrial hyperplasia and cancer in women with PCOS, particularly those who do not receive treatment to induce ovulation or counteract estrogen with progestins [28].
- Tamoxifen Use: Tamoxifen, a selective estrogen receptor modulator commonly used in the treatment and prevention of breast cancer, has estrogenic effects on the endometrium [47]. This can stimulate endometrial cell proliferation and increase the risk of developing atypical hyperplasia and EC [48]. Women on prolonged tamoxifen therapy are monitored closely for endometrial changes [49].
- Nulliparity: Women who have never given birth (nulliparous) are at higher risk of developing EAH and EC [50]. Pregnancy provides periods of progesterone dominance, which protect the endometrium from estrogenic stimulation [51]. Nulliparous women, especially those with chronic anovulation, lack this protective effect and may be exposed to unopposed estrogen for longer periods [52].

### 3.2. Genetic Predisposition

In addition to hormonal factors, certain genetic conditions significantly increase the risk of EAH and its progression to cancer.

- Lynch Syndrome: Lynch syndrome, also known as hereditary nonpolyposis colorectal cancer, is an inherited genetic condition caused by mutations in DNA mismatch repair genes such as *MLH1*, *MSH2*, *MSH6*, and *PMS2* [53,54]. Women with Lynch syndrome are at a significantly elevated risk of developing EC, with a lifetime risk of 40–60% [55]. Often, EAH is a precursor to malignancy in this population. As a result, women with Lynch syndrome are advised to undergo regular checkups and, in some cases, prophylactic hysterectomy to mitigate cancer risk [56].
- Familial Cancer Syndromes: Other less common familial cancer syndromes may also predispose women to EC, including Cowden syndrome (associated with mutations in the *PTEN* gene), which can increase the risk of both breast and EC [57]. These genetic predispositions underscore the importance of genetic testing and counseling for women with a family history of cancer.

### 3.3. Lifestyle and Other Risk Factors

Several lifestyle-associated factors also contribute to the development and progression of EAH as follows.

- Obesity: As mentioned earlier, obesity is one of the most significant risk factors for EAH and EC due to increased estrogen production from adipose tissue [58]. Obesity also promotes a state of chronic low-grade inflammation, which may contribute to cancer development [59]. Additionally, obese women are more likely to have insulin resistance, metabolic syndrome, and other endocrine imbalances that can exacerbate cancer risk [60].
- Sedentary Lifestyle: A lack of regular physical activity is closely linked to obesity, insulin resistance, and hormonal imbalances, all of which elevate the risk of developing atypical hyperplasia and EC [61]. Exercise helps reduce body fat and insulin levels, lowering the estrogen burden thereby reducing cancer risk [62,63].
- Diabetes: Women with diabetes have an increased risk of developing EC, even independent of obesity [64]. Hyperinsulinemia and insulin resistance, common in type 2 diabetes, may promote endometrial proliferation and cancer progression through various mechanisms, including increased levels of insulin-like growth factors, which have mitogenic effects on endometrial cells [65,66].
- Hypertension: Hypertension is often part of the metabolic syndrome, which is closely associated with obesity and diabetes [67,68]. While a direct causal relationship between hypertension and EC remains unclear, hypertension may act as a marker for the constellation of metabolic risk factors, including obesity and insulin resistance, that raise cancer risk [69,70].

## 4. Clinical Presentation and Diagnosis

### 4.1. Symptoms of Endometrial Atypical Hyperplasia

Overview of Symptoms

The most common and often the earliest symptom of EAH is abnormal uterine bleeding (AUB) [71]. This includes a variety of bleeding patterns that deviate from normal menstruation, particularly in premenopausal women, or any uterine bleeding in postmenopausal women.

Abnormal Uterine Bleeding: AUB encompasses heavy, prolonged, or irregular menstrual bleeding in premenopausal women [72]. It may manifest as the following:
  ○ Menorrhagia: heavy or prolonged menstrual periods.
  ○ Metrorrhagia: irregular or intermenstrual bleeding.
  ○ Oligomenorrhea: infrequent menstruation.
  ○ Polymenorrhea: frequent menstrual periods.

Women with chronic anovulation, such as those with PCOS, may experience prolonged periods of no bleeding followed by heavy or prolonged menstruation, often indicating endometrial hyperplasia [73].
Postmenopausal Bleeding: Any bleeding that occurs after a woman has entered menopause is considered abnormal and warrants investigation [74]. Postmenopausal bleeding is a key clinical sign of endometrial hyperplasia or EC, as the endometrium should not be exposed to estrogen stimulation after menopause [75]. Even a single episode of postmenopausal bleeding should prompt diagnostic evaluation.
Other Clinical Signs: Although less common, women with EAH may experience pelvic discomfort or pain. Occasionally, symptoms such as vaginal discharge or spotting between periods may also be present [76].

Because these symptoms overlap with other gynecologic conditions, diagnostic screening is crucial to differentiate EAH from benign conditions or malignancies.

### 4.2. Diagnostic Tools

#### 4.2.1. Endometrial Biopsy

The endometrial biopsy is the gold standard for diagnosing EAH [77]. It involves obtaining a small sample of endometrial tissue, typically through a minimally invasive, outpatient procedure using a suction curette. The tissue is then analyzed histologically for the presence of glandular crowding, nuclear atypia, and other key features of EAH [78]. Studies have shown high sensitivity and specificity for endometrial hyperplasia and carcinoma [79,80]. The Pipelle biopsy technique demonstrated sensitivity up to 97% despite sampling only 4% of the endometrial surface [79]. However, its accuracy may vary depending on the specific pathology, with EAH and EC showing the highest diagnostic accuracy [81]. The Pipelle biopsy demonstrated a positive predictive value for simple EH (42%) and a negative predictive value for EAH (97.6%) [81]. While endometrial biopsy is generally reliable, additional diagnostic methods may be necessary for focal lesions [81]. Novel approaches, such as the GDP-Tao device combining tissue disruption and aspiration, show promising results in obtaining adequate samples for accurate cancer detection [80]. The new device demonstrated a sensitivity of 96% (23/24) and a specificity of 87% (13/15), with a positive predictive value of 92% (23/25) and a negative predictive value of 93% (13/14) [80].

Advantages: It is relatively simple, cost-effective, and can be performed in a clinical setting without the need for general anesthesia [82].
Limitations: Although the biopsy can accurately diagnose hyperplasia and atypia, it may miss focal lesions or small areas of malignancy due to sampling error, particularly in cases of heterogeneous disease [78].

#### 4.2.2. Transvaginal Ultrasound (TVUS)

TVUS is a non-invasive imaging tool commonly used to assess the thickness of the endometrial lining [83]. It provides an initial evaluation of the endometrium, particularly in postmenopausal women, where thickening of the endometrium can indicate hyperplasia or malignancy. Shokouhi et al. reported high accuracy, sensitivity, and specificity for TVS in detecting EH, particularly in postmenopausal women [84]. Similarly, Showkat et al. found TVS to be a sensitive and accurate modality for evaluating EH, with high validity test values [84]. The validity of TVUS in diagnosing EH was assessed, yielding a sensitivity of 94%, specificity of 92%, accuracy of 93%, positive predictive value of 89%, and negative predictive value of 96% [84]. However, Poliakova et al. observed lower sensitivity (51.47% (95%CI 39.03–63.78)) and specificity (59.09% (95%CI 48.09–69.46)) for TVUS in diagnosing EH, suggesting its limited diagnostic value for this specific condition [85]. Metin et al. explored transvaginal sonographic elastography as a potential tool for differentiating between EH and endometrial carcinoma, reporting high sensitivity and specificity [86]. Despite the varying results, TVUS remains a valuable tool for detecting intrauterine pathologies requiring further investigation [85]. Additional research is needed to determine the exact accuracy of TVUS in diagnosing EH [84].

Endometrial Thickness: In postmenopausal women, an endometrial thickness greater than 4–5 mm on TVUS is considered abnormal and warrants further evaluation, as it suggests hyperplasia or cancer [87]. In premenopausal women, the thickness may vary with the menstrual cycle, making it less specific, but a markedly thickened endometrium outside the normal range can still raise suspicion for hyperplasia.
Identifying Abnormalities: TVUS can also detect other endometrial abnormalities, such as polyps or structural changes, which may be contributing to abnormal bleeding [88].

#### 4.2.3. Hysteroscopy

Hysteroscopy is a more advanced diagnostic tool that allows for direct visualization of the uterine cavity [89]. It involves inserting a thin, lighted scope through the cervix into the uterus, providing a detailed view of the endometrial lining. Hysteroscopy is particularly useful in cases where TVUS or biopsy results are inconclusive or when focal lesions such as polyps or localized hyperplasia are suspected [90,91].

Hysteroscopy can also be effective in diagnosing EH, with senior operators correctly predicting over half of the cases [92]. For EH, hysteroscopy showed a sensitivity of 90.4%, a positive predictive value of 58.4%, and a negative predictive value of 86.6% [92]. For benign endometrial lesions, hysteroscopy demonstrated high sensitivity (98.9%) and specificity (97.5%) [93]. However, its performance in detecting EC and EAH is less consistent. One study reported sensitivity and specificity of 76.3% and 93.0% for EC detection [94], while another found 62.5% and 90.8% using a scoring system [95]. A new hysteroscopic scoring system showed improved sensitivity (95.4%) and specificity (98.2%) for EC diagnosis [96]. Despite these variations, hysteroscopy remains a valuable diagnostic tool, especially when combined with biopsy procedures. However, its limitations in excluding EAH and EC emphasize the importance of histopathological confirmation.

Visual Assessment: Hysteroscopy enables clinicians to visually assess the endometrium for abnormalities, such as hyperplasia, polyps, or cancerous lesions, and take targeted biopsies of suspicious areas [71]. It is often considered when other diagnostic tools do not provide sufficient clarity.
Biopsy during Hysteroscopy: The ability to perform targeted biopsies under direct vision improves diagnostic accuracy and reduces the chance of missing focal lesions, such as early-stage cancers that may be missed with blind endometrial biopsy [97].

#### 4.2.4. Differentiating Atypical Hyperplasia from Carcinoma

One of the key challenges in diagnosing EAH is differentiating it from early-stage EC [98,99]. While EAH is considered a precursor to EC, histological and molecular overlap between EAH and well-differentiated (low-grade) EC can make diagnosis challenging [100]. Misclassification can lead to inappropriate management strategies that either over- or under-treat the condition.

- Biopsy Challenges: Endometrial biopsy samples may sometimes be insufficient to differentiate EAH from well-differentiated EC, particularly because both conditions may share features such as glandular crowding and nuclear atypia [101]. Sampling error or focal carcinoma within a background of hyperplasia can complicate diagnosis.
- Hysteroscopy for Clarification: In cases where biopsy results are inconclusive or the pathology report raises suspicion of malignancy, hysteroscopy with directed biopsy can provide additional clarity by targeting abnormal areas that may harbor cancer [102].
- Imaging and Molecular Markers: In some cases, additional diagnostic modalities such as magnetic resonance imaging (MRI) or the evaluation of molecular markers (PTEN, p53) may be used to assess the likelihood of malignant progression [103,104].

## 5. Management of EAH

### 5.1. Conservative vs. Definitive Treatment Approaches

The management of EAH depends largely on the patient’s desire for fertility preservation, overall health, and risk factors for progression to EC. Treatment approaches can be broadly categorized into conservative (hormonal therapy) and definitive (surgical) options.

- Hormonal Therapy:
  ○ Indications: Hormonal therapy is typically indicated for women who wish to preserve their fertility or those for whom surgery is contraindicated due to medical comorbidities [105]. It is also used in younger women or in cases of early-stage disease.
  ○ Progestin Therapy: Progestins, either in oral form or delivered via a levonorgestrel-releasing intrauterine device (LNG-IUD), are the cornerstone of conservative treatment [106]. Progestins counteract the effects of unopposed estrogen, leading to atrophy of the endometrial tissue and regression of hyperplasia [107].
    ▪ Oral Progestins: Commonly prescribed agents include medroxyprogesterone acetate (MPA) and megestrol acetate. These have shown efficacy in reversing hyperplasia, with a response rate of approximately 70–80% [107]. However, oral progestins can be associated with systemic side effects, such as weight gain, mood changes, and thromboembolic events.
    ▪ LNG-IUD: The LNG-IUD has gained popularity as it provides a localized, sustained release of progestin with minimal systemic side effects. Studies have shown that the LNG-IUD implantation can lead to outcomes that are similar or even superior to those achieved by oral progestins, with high rates of disease regression (up to 90%) [107]. It is also well-tolerated and offers long-term protection against hyperplasia.
  ○ Outcomes: While hormonal therapy is effective in many cases, there is a significant risk of recurrence, and the disease may progress to EC if not adequately monitored [7,108]. Complete regression is achieved in most women, but long-term success requires diligent follow-up.
- Hysterectomy:
  ○ Indications: Hysterectomy is the definitive treatment for EAH, especially in women who do not desire future fertility [109]. It is particularly recommended in high-risk cases, including those with recurrent hyperplasia, failure of hormonal therapy, or women with significant comorbidities that increase the risk of cancer development.
  ○ Outcomes: Hysterectomy provides near-total protection against the progression of EAH to EC [109]. In women at high risk, particularly postmenopausal women, or those with concurrent risk factors such as obesity and diabetes, hysterectomy is considered the most reliable treatment option. For women who do not desire children, it is often the preferred course of action.

### 5.2. Fertility-Sparing Options

For women who wish to preserve fertility, conservative management is the primary approach [7].

- Progestin-Based Therapy: As mentioned, both oral progestins and the LNG-IUD are viable options for treating EAH while maintaining fertility [110]. In such cases, close monitoring is essential to ensure disease regression and to prevent progression. Response to progestin therapy is typically monitored by repeat endometrial biopsies every 3–6 months [2].
- Continuous Follow-Up: For fertility-sparing treatment, continuous and rigorous follow-up is critical. Regular endometrial sampling or imaging is necessary to assess for recurrence or progression, which remains a possibility even after apparent initial regression [2]. If progestin therapy fails or hyperplasia recurs, a hysterectomy may be reconsidered after childbearing is complete [108].

### 5.3. Follow-Up and Surveillance

For patients undergoing conservative management, the high risk of recurrence or progression to EC necessitates careful and continuous follow-up.

- Regular Monitoring: Patients on hormonal therapy require frequent follow-up with serial endometrial biopsies every 3–6 months [2]. This allows for the early detection of persistent hyperplasia or progression to carcinoma. If regression is observed, follow-up intervals may be extended.
- Role of Imaging: In addition to biopsies, TVUS can be used to monitor endometrial thickness, although biopsy remains the gold standard for confirming the presence or absence of hyperplasia [111].
- Recurrence Risk: Despite treatment, EAH carries a notable risk of recurrence, particularly in women treated conservatively. The risk of progression to cancer ranges from 20 to 50%, and regular surveillance is critical for identifying these cases early [2]. In patients who have achieved full fertility, hysterectomy is frequently indicated as a definitive measure to eliminate the risk of progression.

## 6. Risk Stratification and Prognosis in EAH

### 6.1. Predicting the Risk of Progression to Endometrial Cancer

EAH is a known precursor to type I EC (endometrioid adenocarcinoma), with a progression risk ranging from 20 to 50%, if untreated. Risk stratification models have been developed to predict which patients are most likely to progress to cancer. These models incorporate a variety of clinical, pathological, and molecular factors.

- Clinical Factors:
  ○ Age: Older women, particularly those who are postmenopausal, have a higher risk of progression from atypical hyperplasia to EC [38]. Postmenopausal women often have additional risk factors such as increased cumulative exposure to estrogen.
  ○ Body Mass Index (BMI): Obesity is a major risk factor for both EAH and EC [112]. Increased adipose tissue leads to higher levels of endogenous estrogen production, contributing to endometrial proliferation without the balancing effect of progesterone [112]. The risk of progression is significantly elevated in obese women.
  ○ PCOS: Women with PCOS often have chronic anovulation, resulting in prolonged exposure to unopposed estrogen, further increasing the risk of hyperplasia progressing to cancer [27,28].
  ○ Unopposed Estrogen Exposure: Women on estrogen replacement therapy without concurrent progestin, those with early menarche or late menopause, and women with nulliparity (no pregnancies) face higher risks due to prolonged estrogen exposure [39].
- Histopathological Features:
  ○ Nuclear Atypia: The presence of nuclear atypia in endometrial hyperplasia is a key predictor of progression to cancer [2]. The more severe the atypia, the higher is the risk.
  ○ Glandular Complexity: Increased glandular crowding and complexity in endometrial tissue can indicate a greater likelihood of malignant transformation [2]. Atypical hyperplasia with cellular architectural complexity is more likely to progress than simple hyperplasia without atypia.
- Risk Models: Several risk models incorporate clinical and histopathological features to predict progression. These models often weigh the significance of factors such as age, BMI, and the presence of nuclear atypia [113]. However, they are not infallible, and their predictive accuracy is limited, requiring continued research on more advanced, molecular-based risk stratification methods [114].

### 6.2. Role of Biomarkers in Risk Stratification

Emerging molecular biomarkers are being studied to improve the risk stratification of patients with EAH [115]. These biomarkers may provide deeper insights into the likelihood of progression to EC, allowing for more personalized treatment approaches [115]. The identification of molecular alterations in endometrial tissue has the potential to refine current risk models.

- *PTEN* Loss:
  ○ *PTEN* is a tumor suppressor gene frequently mutated in ECs, particularly in type I endometrioid adenocarcinoma [104]. Loss of PTEN expression has been identified in 30 to 80% of cases of atypical hyperplasia and is considered a key early event in the transition from hyperplasia to cancer [116].
  ○ The loss of PTEN has been proposed as a marker for increased risk of progression [116]. Studies have shown that women with PTEN loss in endometrial hyperplasia are more likely to develop EC [17]. Research shows that PTEN expression is significantly higher in normal proliferative endometrium and simple hyperplasia compared to complex atypical hyperplasia [117]. However, a meta-analysis found that PTEN loss was not significantly associated with therapy outcomes in EH and EEC treated with progestins [118]. Recent studies have demonstrated that loss of PTEN expression may reflect progression to endometrial carcinoma, with negative PTEN immunoexpression indicating poor prognosis and higher recurrence probability [119]. Additionally, PTEN expression is downregulated in atypical hyperplastic and neoplastic endometrial tissues, showing an inverse relationship with tumor grade, stage, and myometrial invasion [120]. These findings suggest that PTEN may have potential as a screening tool for precancerous endometrial lesions.
  ○ This biomarker may be particularly useful in identifying high-risk patients who might benefit from more aggressive treatment, such as hysterectomy, even in the absence of severe clinical or histological risk features.
- *p53* Mutations:
  ○ The *p53* gene is another critical tumor suppressor frequently mutated in various cancers, including EC [121]. Mutations in *p53* are more commonly associated with type II serous ECs, but aberrant *p53* expression has also been detected in a subset of endometrioid adenocarcinomas [122].
  ○ While *p53* mutations are less common in atypical hyperplasia, their presence may indicate a more aggressive disease course [123]. Some studies suggest that *p53* abnormalities could serve as a biomarker for progression in hyperplasia cases, especially when detected alongside other molecular changes such as *PTEN* loss or *KRAS* mutations.
  ○ P53 pathway markers, including p21, mdm2, and phospho-p63, have shown promise in refining molecular classifications and predicting clinical outcomes [124]. Expression of estrogen and progesterone receptors correlates with simple EH and well-differentiated tumors, while p53 and Ki-67 overexpression indicates a more malignant phenotype [125]. Positive expression of p53 in endometrial hyperplasia may indicate progression to carcinoma [126]. Elevated p53 expression was associated with poor differentiation in EC. P53 expression was significantly higher in cases with FIGO stages III and IV compared to stages I and II (100% vs. 18.1%, *p* = 0.0016) and in grade 3 tumors compared to grades 1 and 2 (50% vs. 0%, *p* = 0.0116) [127]. These findings suggest that molecular markers can improve risk stratification and guide personalized treatment approaches in endometrial pathologies.
- Microsatellite Instability:
  ○ MSI is a hallmark of defective DNA mismatch repair (MMR) and is observed in approximately 20–30% of patients with ECs [128]. MMR defects and MSI can occur early in the progression from hyperplasia to carcinoma, particularly in women with Lynch syndrome, a hereditary cancer syndrome [53].
  ○ The presence of MSI in endometrial hyperplasia could be a significant predictor of progression, particularly in patients with known genetic predispositions such as Lynch syndrome. Screening for MSI or MMR deficiency in patients with atypical hyperplasia could help identify those at higher risk for developing EC [129].
  ○ Studies have shown that EH, particularly EAH, often exhibits MSI and loss of MMR protein expression, which are precursors to endometrial carcinoma [130,131]. The progression from EAH to carcinoma is associated with an increase in unstable microsatellite loci and tumor mutational burden [131]. MLH1 promoter methylation is an early event in this process, although it may not be required for MLH1 silencing and MMR loss [132]. MSI analysis of EAH in young patients (≤50 years) may serve as a prognostic marker for potential progression to MSI-high endometrial carcinomas [133]. Combined MSI and immunohistochemistry analysis can help identify hereditary nonpolyposis colorectal cancer patients among young women with EC and EAH [133].
- *KRAS* Mutations:
  ○ The *KRAS* oncogene is another genetic alteration found in both endometrial hyperplasia and carcinoma [134]. *KRAS* mutations are involved in cellular proliferation and differentiation and have been detected in up to 20% of cases of atypical hyperplasia [135].
  ○ *KRAS* mutations are associated with type I EC and may be involved in early carcinogenesis [18]. Studies have found *KRAS* mutations in both cancerous and non-cancerous endometrial tissues, suggesting their potential as early indicators of malignancy risk [136]. Other biomarkers, such as *PTEN* loss, increased stromal *p16* expression, and decreased *PAX2* expression, have been associated with the transition from EAH to EC [137]. *KRAS* mutation is common in endometrial cancer and may be involved in disease progression, but its role in fertility-sparing treatment outcomes is not yet known [134].
  ○ While *KRAS* mutations alone do not necessarily predict poor outcomes, their presence alongside other mutations, such as *PTEN* loss, may indicate a higher risk of progression to carcinoma [16]. Further studies are needed to determine the prognostic significance of *KRAS* mutations in EAH.

### 6.3. Prognostic Implications of Biomarkers

Incorporating molecular markers such as *PTEN* loss, *p53* mutations, MSI, and *KRAS* mutations into traditional risk models could improve the accuracy of predicting progression to EC [138]. These biomarkers offer the potential for more tailored treatment strategies.

- High-Risk Patients: Those identified with molecular abnormalities such as *PTEN* loss or MSI may benefit from early definitive treatment, such as hysterectomy, even if they have not yet developed overt carcinoma [139].
- Low-Risk Patients: Women without significant molecular changes could be candidates for more conservative management, such as progestin therapy, with close follow-up, reducing the need for aggressive interventions in all cases of atypical hyperplasia.

## 7. EAH in Special Populations

### 7.1. Premenopausal Women

Premenopausal women with EAH face unique challenges, particularly those related to fertility preservation and the management of comorbidities such as PCOS. Unlike postmenopausal women, younger patients may prioritize fertility-sparing options, necessitating a more conservative approach to treatment.

- Fertility-Preserving Options:
  ○ For women of reproductive age, especially those desiring future pregnancies, conservative management is preferred. The standard treatment involves progestin-based therapy, either through oral progestins or the use of an LNG-IUD [140].
  ○ Oral progestins such as MPA and megestrol acetate are commonly prescribed for premenopausal women [108]. Progestins help counterbalance the effects of unopposed estrogen and lead to regression of hyperplasia [2]. However, these women require frequent follow-up with repeat biopsies to monitor for regression or progression.
  ○ LNG-IUD: For fertility preservation, the LNG-IUD is often favored because of its local effect on the endometrium and lower systemic side effects [141]. The LNG-IUD has been shown to be highly effective, with response rates reaching up to 90% in premenopausal women.
  ○ If progestin therapy successfully leads to regression, patients can attempt pregnancy [142]. However, once childbearing is complete, definitive treatment such as hysterectomy may be considered to eliminate the risk of progression to EC.
- Management of Coexisting PCOS:
  ○ PCOS is a common comorbidity in premenopausal women with EAH, contributing to prolonged anovulation and unopposed estrogen exposure [143]. In these women, treating the underlying hormonal imbalance is critical.
  ○ Weight management and metformin may be considered to improve insulin resistance and reduce the hyperandrogenic state seen in PCOS [144,145]. Additionally, combined oral contraceptives can help regulate menstrual cycles and reduce estrogen-driven endometrial proliferation in women with mild hyperplasia without atypia [146].
  ○ For women with atypical hyperplasia and PCOS, progestin-based therapy remains the cornerstone of treatment, but the underlying metabolic and hormonal imbalances should be addressed to reduce future risks [147].
- Surveillance:
  ○ Continuous follow-up is essential, including regular endometrial biopsies every 3–6 months [109]. While fertility-sparing treatment can be effective, the risk of recurrence or progression remains significant, and patients must be counseled on the need for long-term monitoring.

### 7.2. Postmenopausal Women

Because of the risk of progression to EC and the lack of concern for fertility preservation in postmenopausal women, EAH is managed differently. In addition, HT might complicate management considerations.

- Higher Risk of Progression:
  ○ Postmenopausal women are at a substantially increased risk of progressing from EAH to EC, with studies showing up to a 50% progression rate in untreated cases [148]. This is due in part to the absence of regular menstrual shedding and the prolonged, often unopposed exposure to estrogen in women with obesity or those on estrogen-only HT [149].
  ○ Definitive treatment in the form of a hysterectomy is typically recommended for postmenopausal women, especially those who are high-risk or have comorbidities such as obesity, diabetes, or hypertension [150]. Hysterectomy offers definitive protection against progression and is often the preferred approach once the diagnosis of atypical hyperplasia is confirmed.
- Implications of Hormone Therapy:
  ○ Postmenopausal women on estrogen-only HT are at an increased risk of developing EAH and EC due to unopposed estrogen stimulation of the endometrium [39]. The addition of progestins to HT (combined HT) in women with an intact uterus is essential to reduce this risk [39].
  ○ Women diagnosed with EAH while on HT may need to discontinue estrogen-only therapy or switch to combined HT, which includes a progestin component to counteract the effects of estrogen on the endometrium.
  ○ In some cases, women who cannot tolerate combined HT may be candidates for LNG-IUD placement, which provides localized progestin to protect the endometrium from hyperplasia while allowing for systemic estrogen therapy [151].
- Optimal Management Strategies:
  ○ Hysterectomy remains the most reliable and often preferred option for postmenopausal women with atypical hyperplasia, as it eliminates the risk of progression and recurrence [152]. Women who are poor surgical candidates due to medical comorbidities may be treated with progestin-based therapy, but the risk of recurrence and progression remains higher than that associated with surgical management [153].
  ○ For women who opt for conservative management, continuous follow-up with serial endometrial biopsies or TVUS is mandatory. However, long-term conservative management is not ideal for most postmenopausal women due to their higher cancer risk.
- Surveillance:
  ○ Postmenopausal women on conservative treatment should undergo frequent endometrial surveillance, similar to premenopausal women [154]. However, the threshold for transitioning to hysterectomy is generally lower in this population due to their elevated risk of cancer progression.

## 8. Conclusions

**Figure 1:**
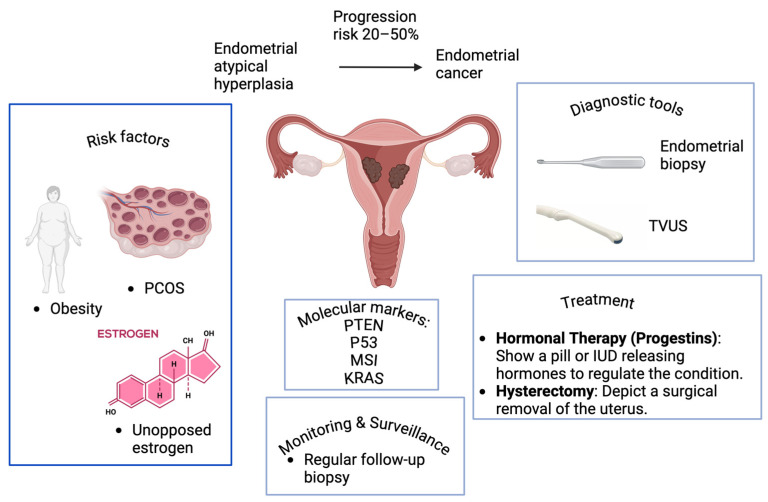
Overview of endometrial atypical hyperplasia and its progression to endometrial cancer. PCOS: polycystic ovarian syndrome, TVUS: transvaginal ultrasound, MSI: microsatellite instability, IUD: intrauterine device.

EAH is a significant precursor to EC, particularly type I endometrioid adenocarcinoma, with progression rates as high as 50% in untreated cases. The strong relationship between EAH and EC emphasizes the need for timely diagnosis and appropriate treatment to prevent malignant transformation. Key risk factors such as unopposed estrogen exposure, obesity, PCOS and genetic predispositions such as Lynch syndrome further increase the likelihood of progression. Management strategies must be carefully tailored based on individual risk factors, including patient age, reproductive desires, and comorbidities. While hysterectomy remains the definitive treatment for high-risk patients, particularly in postmenopausal women, fertility-sparing options such as progestin therapy and LNG-IUDs provide effective alternatives for premenopausal women (Figure 1).

Looking forward, the future of EAH management will likely be shaped by advances in personalized medicine, which involves assessing individual molecular profiles to guide treatment. Emerging molecular-targeted therapies that inhibit pathways such as PI3K/AKT/mTOR offer promise for patients at higher risk or those unsuitable for surgery. In addition, improvements in diagnostic tools, such as liquid biopsies and ctDNA monitoring, could enable earlier detection and better surveillance, reducing the need for invasive biopsies. These innovations, combined with a more nuanced understanding of molecular risk factors, will help to create individualized treatment plans that balance the need for effective cancer prevention with the patient’s personal health and fertility goals.

## Figures and Tables

**Table 1:** The search strategies outline.

Items	Specification
Timeframe	To 31 August 2024
Database	PubMed
Search terms used	“endometrial atypical hyperplasia”, “endometrial cancer”
Inclusion and exclusion criteria	All references were SCI-indexed articles The language is English
Selection process	Two independent reviewers evaluated the titles and abstracts to determine eligibility.

## Data Availability

Not applicable.
